# Determination of the discriminating concentration of chlorfenapyr (pyrrole) and *Anopheles gambiae *sensu lato susceptibility testing in preparation for distribution of Interceptor® G2 insecticide-treated nets

**DOI:** 10.1186/s12936-021-03847-3

**Published:** 2021-07-14

**Authors:** Richard M. Oxborough, Aklilu Seyoum, Yemane Yihdego, Joseph Chabi, Francis Wat’senga, Fiacre R. Agossa, Sylvester Coleman, Samdi Lazarus Musa, Ousmane Faye, Michael Okia, Mohamed Bayoh, Evelyne Alyko, Jean-Desire Rakotoson, Hieronymo Masendu, Arthur Sovi, Libasse Gadiaga, Bernard Abong’o, Kevin Opondo, Ibrahima Baber, Roch Dabire, Virgile Gnanguenon, Gedeon Yohannes, Kenyssony Varela, Etienne Fondjo, Jenny Carlson, Jennifer S. Armistead, Dereje Dengela

**Affiliations:** 1 PMI VectorLink Project, Abt Associates, 6130 Executive Blvd, Rockville, MD 20852 USA; 2 Entomology Department, National Institute of Biomedical Research, Avenue de la Démocratie, Kinshasa, Democratic Republic of the Congo; 3 PMI VectorLink Project, Abt Associates, Kinshasa, Democratic Republic of the Congo; 4 PMI VectorLink Project, Abt Associates, Plot 11 Waterson Road, Fuo, Tamale, Ghana; 5 PMI VectorLink Project, Abt Associates, Gte No. 12 TOS Benson Crescent, Utako, Abuja, Nigeria; 6 Département de Biologie Animale, Université Cheikh Anta Diop, Bp 5005 Dakar-Fann, Dakar, Senegal; 7 PMI VectorLink Project, Abt Associates, Tororo, Uganda; 8 PMI VectorLink Project, Abt Associates, Njoka Road, Off Kwacha Road, Olympia, Box 39090, Lusaka, Zambia; 9 PMI VectorLink Project, Abt Associates, Freetown, Sierra Leone; 10 PMI VectorLink Project, Abt Associates, Lot Ex La Sice, Ambalanaomby, Farafangana, Madagascar; 11 PMI VectorLink Project, Abt Associates, 1 Pascoe Avenue, Belgravia, Harare, Zimbabwe; 12 PMI VectorLink Project, Abt Associates, Cite du Niger 1, Rue 30, Porte 612, Bamako, Mali; 13 Faculty of Infectious and Tropical Diseases, Disease Control Department, London School of Hygiene and Tropical Medicine, London, WC1E 7HT UK; 14 Faculty of Agronomy, University of Parakou, BP 123, Parakou, Benin; 15 PMI VectorLink Project, Abt Associates, Whitehouse, Milimani, Kisumu, Ojijo Oteko Road, P.O. Box 895-40123, Kisumu, Milimani Kenya; 16 PMI VectorLink Project, Abt Associates, 16th Street, Beach Side, Sinkor, Monrovia Liberia; 17 Institute of Health Science Research, Malaria and Tropical Neglected Research Unit, 01 BP 545, Bobo-Dioulasso, Burkina Faso; 18 PMI VectorLink Project, Abt Associates, Plot 28 Avenue Pierre Ngendandumwe, Bujumbura, Burundi; 19 PMI VectorLink Project, Abt Associates, Gerje Rood Sami Building, Floor 1, Office no 105, P.O. Box : 13646, Addis Ababa, Ethiopia; 20 PMI VectorLink Project, Abt Associates, Rua Justino Chemane, No. 237 Sommerschield 2, Maputo, Mozambique; 21 PMI VectorLink Project, Abt Associates, P.O. Box 14 025, Mballa II, Dragages, P.O. Box 14 025, Yaounde, Cameroon; 22 U.S. President’s Malaria Initiative, U.S. Agency for International Development, Washington, DC USA

**Keywords:** Chlorfenapyr, Pyrrole, *Anopheles gambiae*, Interceptor G2, CDC bottle bioassay, Discriminating concentration, Insecticide-treated net, Insecticide resistance

## Abstract

**Background:**

Following agricultural use and large-scale distribution of insecticide-treated nets (ITNs), malaria vector resistance to pyrethroids is widespread in sub-Saharan Africa. Interceptor® G2 is a new dual active ingredient (AI) ITN treated with alpha-cypermethrin and chlorfenapyr for the control of pyrethroid-resistant malaria vectors. In anticipation of these new nets being more widely distributed, testing was conducted to develop a chlorfenapyr susceptibility bioassay protocol and gather susceptibility information.

**Methods:**

Bottle bioassay tests were conducted using five concentrations of chlorfenapyr at 12.5, 25, 50, 100, and 200 µg AI/bottle in 10 countries in sub-Saharan Africa using 13,639 wild-collected *Anopheles gambiae *sensu lato (*s.l*.) (56 vector populations per dose) and 4,494 pyrethroid-susceptible insectary mosquitoes from 8 colonized strains. In parallel, susceptibility tests were conducted using a provisional discriminating concentration of 100 µg AI/bottle in 16 countries using 23,422 wild-collected, pyrethroid-resistant *An. gambiae s.l.* (259 vector populations). Exposure time was 60 min, with mortality recorded at 24, 48 and 72 h after exposure.

**Results:**

Median mortality rates (up to 72 h after exposure) of insectary colony mosquitoes was 100% at all five concentrations tested, but the lowest dose to kill all mosquitoes tested was 50 µg AI/bottle. The median 72-h mortality of wild *An. gambiae s.l.* in 10 countries was 71.5, 90.5, 96.5, 100, and 100% at concentrations of 12.5, 25, 50, 100, and 200 µg AI/bottle, respectively. Log-probit analysis of the five concentrations tested determined that the LC_95_ of wild *An. gambiae* s*.l.* was 67.9 µg AI/bottle (95% CI: 48.8–119.5). The discriminating concentration of 203.8 µg AI/bottle (95% CI: 146–359) was calculated by multiplying the LC_95_ by three. However, the difference in mortality between 100 and 200 µg AI/bottle was minimal and large-scale testing using 100 µg AI/bottle with wild *An. gambiae s.l.* in 16 countries showed that this concentration was generally suitable, with a median mortality rate of 100% at 72 h.

**Conclusions:**

This study determined that 100 or 200 µg AI/bottle chlorfenapyr in bottle bioassays are suitable discriminating concentrations for monitoring susceptibility of wild *An. gambiae s.l*., using mortality recorded up to 72 h. Testing in 16 countries in sub-Saharan Africa demonstrated vector susceptibility to chlorfenapyr, including mosquitoes with multiple resistance mechanisms to pyrethroids.

**Supplementary Information:**

The online version contains supplementary material available at 10.1186/s12936-021-03847-3.

## Background

The core vector control interventions recommended by the World Health Organization (WHO) to reduce malaria transmission are universal coverage with insecticide-treated nets (ITN) and/or indoor residual spraying (IRS) of houses [1]. An estimated 1.9 billion ITNs were delivered by manufacturers to countries in sub-Saharan Africa between 2004 and 2019, with 213 million ITNs distributed in 2019 alone [2]. Between 2000 and 2015 it is estimated that vector control averted 663 million clinical cases of malaria in sub-Saharan Africa, with ITNs contributing to 68% of that reduction [3]. Pyrethroid insecticides remain the dominant chemical class used on ITNs due to their low cost (< $2 per net), low human toxicity and efficacy against mosquitoes through rapid knock-down, mortality and repellency [4, 5]. Currently there are 15 standard pyrethroid net products that have WHO prequalification (PQ) listing, consisting of seven that contain alpha-cypermethrin, seven deltamethrin and one permethrin [6]. Following agricultural use and large-scale distribution of pyrethroid ITNs, resistance to pyrethroids in sub-Saharan Africa is widespread, with many countries reporting high resistance intensity, which is likely to lead to vector control failure [7–11].

To manage insecticide resistance and effectively control malaria vectors, it is important for ITNs to use insecticides with different modes of action. New ‘dual active ingredient’ nets treated with two different active ingredients (AIs) have been developed, although to date these all include a pyrethroid as one of the AIs. Examples include Interceptor G2 (treated with chlorfenapyr and alpha-cypermethrin) and Royal Guard (treated with pyriproxyfen and alpha-cypermethrin) ITNs, which received WHO PQ listing in 2018 and 2019, respectively [6]. Experimental hut studies of Interceptor G2 ITNs have shown particular promise, with high efficacy and wash durability against pyrethroid-resistant malaria vectors demonstrated in Benin, Burkina Faso and Côte d’Ivoire [12–14]. Chlorfenapyr is a pyrrole compound with a non-neurotoxic mode of action that involves uncoupling of oxidative phosphorylation via disruption of the proton gradient [15, 16]. This uncoupling at the mitochondria ultimately results in disruption of ATP (Adenosine 5'-triphosphate) production, cellular death and organism mortality [17]. Chlorfenapyr is a pro-insecticide, meaning that after uptake by the insect the parent form of chlorfenapyr (CL303630) is metabolized by cytochrome P450 enzymes into the active metabolite (CL303268) [16]. Chlorfenapyr is used as a termiticide and in agriculture as a foliar-applied insecticide to control insect and mite pests of various fruit, vegetable, grain, herb, spice, and tea crops but is not yet widely used in sub-Saharan Africa and is fairly new for vector control [18].

As of 2020 there was no published guidance regarding chlorfenapyr susceptibility test procedures or discriminating concentration. Insecticide susceptibility tests of malaria vectors are normally conducted using either pre-treated filter papers that are prepared by a WHO collaborating institution (Universiti Sains, Malaysia) and distributed to field sites for use in tube tests or by using bottle bioassay procedures according to US Centers for Disease Control and Prevention (CDC) defined discriminating concentrations [19, 20]. This delay in guidance was partly due to the non-neurotoxic nature of chlorfenapyr meaning that standard WHO testing protocols needed adaptation. WHO initially proposed 5% chlorfenapyr filter papers with silicon oil for susceptibility testing [21], but this methodology was not taken forward in multi-centre studies coordinated by WHO.

The US President’s Malaria Initiative (PMI) funding supports regular insecticide resistance monitoring in partnership with national malaria control programmes (NMCPs) in sub-Saharan Africa to assist with national vector control decision-making. In anticipation of Interceptor G2 nets being distributed in sub-Saharan Africa, chlorfenapyr susceptibility testing using a modified bottle bioassay protocol was conducted to determine a suitable discriminating concentration and to gather baseline susceptibility information.

## Methods

### Study sites and chlorfenapyr dosages tested

**Fig. 1:**
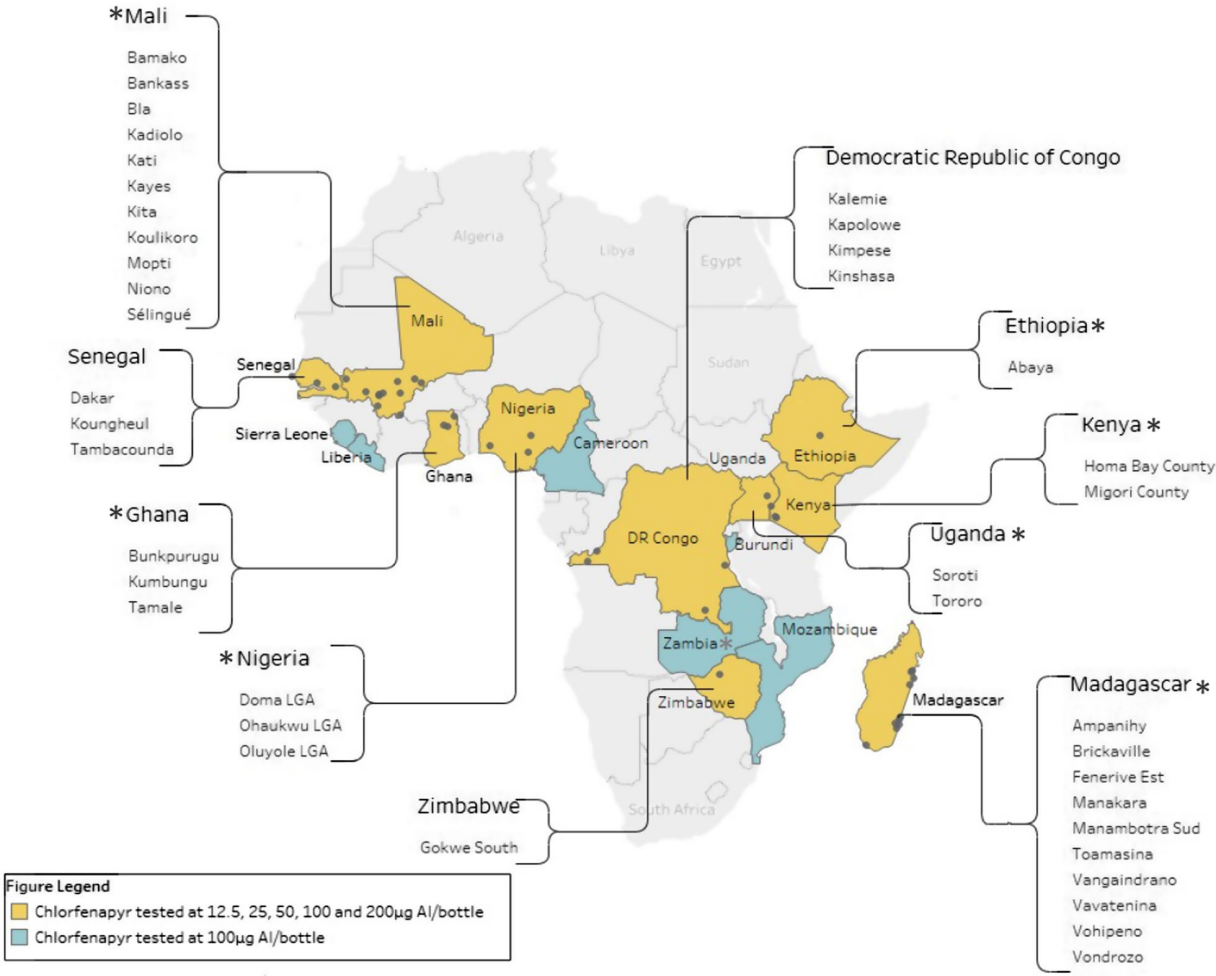
Locations of insecticide susceptibility testing sites where mortality of wild *Anopheles gambiae s.l.* was measured in bioassays following exposure to a full (yellow) or limited (blue) range of concentrations of chlorfenapyr. *denotes countries where a susceptible insectary strain was also tested with the full range of concentrations

Experiments were conducted using five concentrations of chlorfenapyr at 12.5, 25, 50, 100, and 200 µg AI/bottle. This narrow range of doses was chosen based on earlier tests conducted by CDC. Tests were conducted on wild uncharacterized *An. gambiae s.l.* in 10 countries in sub-Saharan Africa, out of which 8 countries conducted additional tests with colonized pyrethroid-susceptible strains. The countries included: The Democratic Republic of Congo (4 sites), Ethiopia (1 site), Ghana (3 sites), Kenya (2 sites), Madagascar (10 sites), Mali (11 sites), Nigeria (3 sites), Senegal (3 sites), Uganda (2 sites), and Zimbabwe (1 site). Locations are shown in Fig. 1. In parallel, susceptibility tests were conducted using a provisional discriminating concentration of 100 µg AI/bottle in 16 countries. This concentration was chosen based on preliminary bottle bioassay testing by CDC, which established a provisional discriminating concentration of 100 µg AI/bottle (Dr WG Brogdon, 2017, personal communication). All bioassays were conducted between 2017 and 2020.

### Preparation of solutions

Treatment of 250-ml Wheaton® bottles was conducted locally in the country of testing using technical grade chlorfenapyr dissolved in acetone. A vial containing 5 g of technical grade (99.9% pure) chlorfenapyr was supplied by BASF (Ludwigshafen, Germany) to each country team and a stock solution was prepared at 1 mg/ml by weighing 100 mg and dissolving with 100 ml acetone. The stock solution was prepared in an amber glass bottle (or clear glass bottle covered with aluminium foil) to avoid exposure to UV light and sealed with a tightly fitting lid to prevent evaporation before being stored at 4 °C in a refrigerator for a maximum of 3 months. A test solution of 200 µg/ml was prepared by performing a five times dilution by mixing 10 ml of the stock solution with 40 ml of acetone. Diluents were serially prepared with twofold dilutions of 100 µg/ml, 50 µg/ml, 25 µg/ml, and 12.5 µg/ml. Stock solution of chlorfenapyr in acetone were warmed to room temperature before conducting dilution.

### Bioassay procedures

Glass bottles and plastic caps were cleaned using detergent solution before being rinsed thoroughly with water and left overnight to dry. Each 250-ml glass bottle and its plastic cap were coated with 1 ml of insecticide solution by rolling and inverting the bottles according to CDC procedures until all visible signs of liquid were gone [20]. In parallel, a negative control bottle was coated with 1 ml of acetone. All bottles were dried overnight in the dark with bottle caps off and bioassays were conducted within 24 h of treating bottles. In general, a total of 80–100 female mosquitoes, aged 2 to 5 days old, were exposed for 60 min in four replicates of 20–25 mosquitoes, with an additional single replicate of 25 mosquitoes used for the negative control (bottle treated with 1 ml acetone). A total of 13,639 wild-collected *An. gambiae s.l.* (56 vector populations per dose) in 10 countries were tested using five concentrations of chlorfenapyr. While a total of 4,494 pyrethroid-susceptible insectary mosquitoes from eight colonized strains were tested. A total of 23,422 wild-collected, pyrethroid-resistant *An. gambiae s.l.* (259 vector populations) were tested at the discriminating concentration of 100 µg AI/bottle in 16 countries. After exposure, mosquitoes were transferred to clean paper cups and provided with 10% sugar solution. Mortality was recorded at the end of the 60-min exposure and at 24, 48 and 72 h after exposure. Tests were conducted during the day time with effort made to keep testing and holding conditions within WHO guidelines of 27 °C ± 2 °C and relative humidity of 75% ± 10% [19]. Temperature and humidity were monitored and recorded, however, in several cases could not be accurately controlled, as tests with wild-collected mosquitoes were generally conducted in improvised field insectaries which did not have robust temperature and humidity controls.

### Mosquito species tested

Insectary-reared, pyrethroid-susceptible colonies of *An. gambiae *sensu stricto (*s.s*.) Kisumu strain were used for testing in six countries (Ghana, Kenya, Madagascar, Nigeria, Uganda, Zambia) while *Anopheles coluzzii* Ngousso strain was used in Mali and *Anopheles arabiensis* Adama strain in Ethiopia. Larval collections of wild *An. gambiae s.l.* were made in areas where pyrethroid resistance had previously been detected from temporary sunlit pools between 2017 and 2020 (timing varied by country) using larval dippers. Larvae were subsequently transported to a field insectary where they were reared in water collected from the field and fed with Tetramin® fish food. Emerging adult mosquitoes were provided with cottonwool pads dipped in 10% sugar solution until they were used in insecticide susceptibility tests. Wild *Anopheles* were identified morphologically as *An. gambiae s.l.* in all 16 countries using the key of Gillies and Coetzee [22]. Molecular analysis to determine species of these test mosquitoes was not conducted. However, *An. gambiae s.l.* collected from the same locations for other purposes were identified to species by PCR using the protocols of either Scott, Santolamazza, or Wilkins to determine members of the *An. gambiae* species complex [23–26].

### Data analysis

Insecticide susceptibility results were presented as unadjusted percentage mortality at the end of 60 min and subsequently 24, 48 and 72 h after bioassay exposure. If negative control mortality was greater than 20%, the data were discarded, and tests were repeated. Box plots are used to present mortality data showing the median and interquartile range, with whiskers representing one and a half times the interquartile range and small circles outside the whiskers considered outliers. PoloPlus (LeOra Software, Parma MO, USA) was used to conduct probit analysis on the logarithmic scale to calculate the concentration of chlorfenapyr needed to kill a defined proportion of mosquitoes, known as lethal concentration (LC). Mortality data (72 h after exposure) was included for each concentration used in the analysis (12.5, 25, 50, 100, 200 µg AI/bottle) to determine the LC_50_, LC_95_ and LC_99_ (concentration needed to achieve 50, 95 and 99% mortality) for wild *An. gambiae s.l.* The LC_95_ value was then multiplied by three to give a discriminating concentration (LC_95_ × 3 = DC) as described by Lees et al. [27]. The WHO approach of multiplying the LC_99_ by two was also used to determine a discriminating concentration [28]. Probit analysis was not conducted with data generated using insectary strains as there was not a sufficient spread of data to fit the probit curve.

## Results

**Fig. 2:**
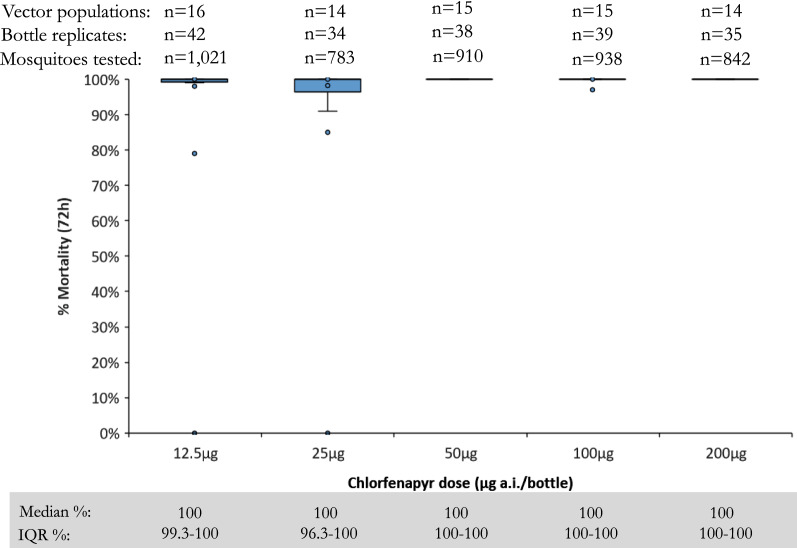
Percentage mortality (72 h) after exposure to each of five concentrations (12.5, 25, 50, 100, 200 µg AI/bottle) of chlorfenapyr in bottle bioassays using pyrethroid susceptible colony mosquito strains in eight countries

Median mortality rates of pyrethroid-susceptible colony strains across eight countries were 100% at 72 h post-exposure to each of five chlorfenapyr concentrations tested in bottle bioassays, with 50 µg AI/bottle being the lowest concentration to kill every mosquito tested (Fig. 2).

**Fig. 3:**
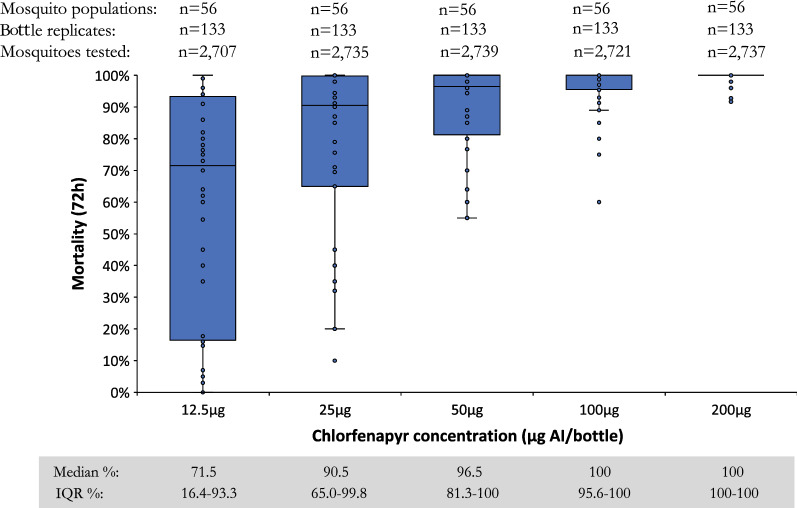
Median percentage mortality of wild *Anopheles gambiae s.l.* 72 h after exposure to chlorfenapyr at concentrations of 12.5, 25, 50, 100, and 200 µg AI/bottle in bottle bioassay in 10 countries

A clear positive response with increasing mean mortality rates at every chlorfenapyr concentration was observed among wild *An. gambiae s.l.* (Fig. 3). The median 72-h mortality was 71.5, 90.5, 96.5, 100, and 100% at 12.5, 25, 50, 100, and 200 µg AI/bottle, respectively. Log-probit analysis determined the LC50 as 7.7 µg AI/bottle (95% confidence interval (CI): 5.5–9.8), LC95 as 67.8 µg AI/bottle (95% CI: 55.2–89.4), and LC99 as 166.9 µg AI/bottle (95% CI: 120.4–266.5). The discriminating concentration was calculated at either 203.4 µg AI/bottle (95% CI: 166–268) using the method of Lees et al*.* [27] or 333.8 µg AI/bottle (95% CI: 241–533) using the WHO approach [28].

**Fig. 4:**
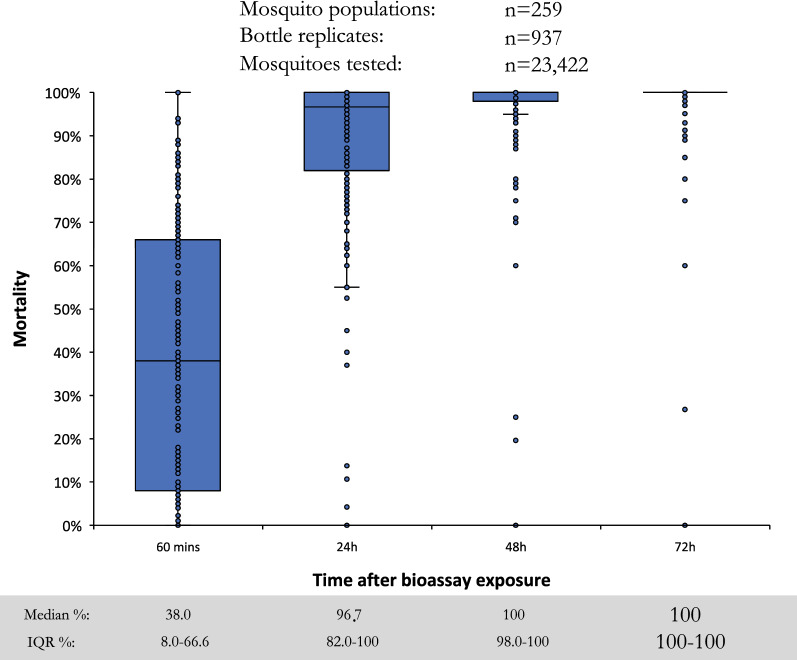
Percentage mortality of wild *Anopheles gambiae s.l.* 60 min, 24 h, 48 h, and 72 h after exposure to the provisional discriminating concentration of chlorfenapyr at 100 µg AI/bottle in 16 countries

**Fig. 5:**
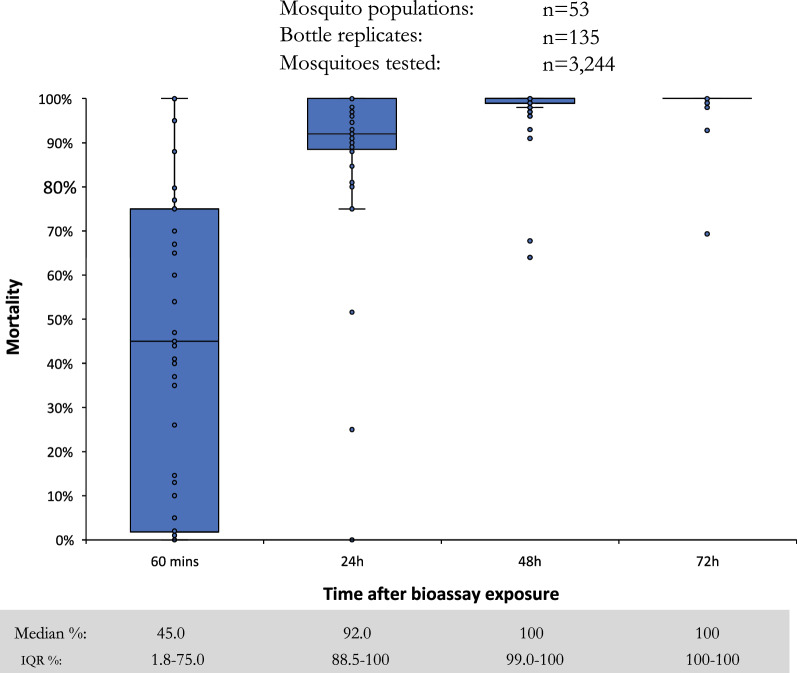
Percentage mortality of wild *Anopheles gambiae s.l.* 60 min, 24 h, 48 h, and 72 h after exposure to chlorfenapyr at 200 µg AI/bottle in 10 countries

At the provisional discriminating concentration of 100 µg AI/bottle, large variation in per cent mosquitoes knocked-down at 60 min was observed (Fig. 4), although the median value was low at 38.0% (interquartile range (IQR): 8.0–66.6%), demonstrating the slow acting nature of pyrrole insecticides compared to pyrethroids [29]. Results confirmed that a holding time of 72 h post-exposure is required, with median mortality of 96.7% (IQR: 82.0–100) at 24 h compared to 100% (IQR: 100–100) at 72 h. While the median mortality was 100% after 72 h, there were many outliers when mortality was < 98%, indicating that 100 µg AI/bottle may not be a suitable discriminating concentration. Tests conducted with 200 µg AI/bottle in 10 countries produced similar trends to the 100 µg concentration, reaching a median of 100% (IQR: 100–100) mortality at 72 h (Fig. 5).

In this study it was not always possible to closely regulate testing and holding temperature however, the temperature was consistently < 25 °C in only Madagascar and Zimbabwe. However, it should be noted that in Madagascar mortality was < 98% with 100 or 200 µg AI/bottle in seven sites where testing and holding temperatures were regularly below the WHO-recommended range of 27 ± 2 °C. Molecular species identification indicated that the predominant species tested in the dose-ranging studies were *An. gambiae* in DR Congo and Madagascar, *An. coluzzii* in Mali, mixed *An. gambiae*/*coluzzii* in Ghana and Nigeria, and *An. arabiensis* in Ethiopia, Kenya, Senegal and Uganda (Additional file 1: Table S1). All wild malaria vectors used for chlorfenapyr susceptibility tests were found to be resistant to pyrethroid insecticides, except for a few locations in Madagascar (Additional file 1: Table S1). Raw bioassay results are available in Additional file 2: database 1 (insectary strains tested with 12.5, 25, 50, 100 and 200 μg AI/bottle), Additional file 3: database 2 (wild *An. gambiae s.l.* tested with 12.5, 25, 50, 100 and 200 μg AI/bottle) and in Additional file 4: database 3 (wild *An. gambiae s.l.* tested with 12.5, 25, 50, 100 and 200 μg AI/bottle.

## Discussion

In this study, susceptibility tests conducted with a pyrethroid-susceptible colony and wild *Anopheles* species confirmed that chlorfenapyr is a slower-acting insecticide when compared with neurotoxic pyrethroids [29]. While pyrethroids are characterized by rapid knock-down of susceptible mosquitoes within a few minutes, chlorfenapyr-induced knock-down at 60 min post-exposure was generally low (albeit highly variable). Despite the low levels of knock-down, more than 90% of chlorfenapyr-induced mortality occurred within 24 h of exposure, but 72 h holding period was required to consistently reach > 98% mortality at 100 and 200 µg AI/bottle. It has previously been shown that 25 µg AI/bottle was sufficient to kill 100% of a susceptible colonized strain of *An. arabiensis* but mortality rates were significantly lower with wild *An. arabiensis* with 100 µg and 200 µg AI/bottle producing mortality > 98% [30]. Results presented here are in keeping with this trend, with pyrethroid-susceptible colony strains killed at lower concentrations than wild *An. gambiae s.l*. Inbreeding over a period of several decades reduces the overall fitness of reference strains, therefore it is important to conduct testing against wild mosquitoes before widespread use for vector control [31]. Other factors which may contribute to higher toxicity with insectary colonies in bioassays could be related to the mode of action which is intertwined with mosquito metabolism [32]. The circadian rhythm of colonies can be different to wild *Anopheles* populations either due to rearing under reverse photoperiod or due to daytime blood feeding, which would result in greater metabolic activity during daytime [33, 34]. Therefore, there could be greater impact of the insecticide on insectary-colonized mosquitoes with daytime exposures because they are more active during the day than wild mosquitoes that are more active during the night under natural conditions.

Others have proposed a discriminating concentration of 50 µg AI/bottle based on bottle bioassays performed with susceptible *An. gambiae* Kisumu strain and wild collected *An. arabiensis*, *An. gambiae s.s.* and *Anopheles funestus* from western Kenya [35]. In this study, 50 µg AI/bottle was sufficient against susceptible-colony mosquitoes but mean mortality of wild *An. gambiae s.l.* across 10 countries was only 93% (95% CI: 86.3–99.5) after 72 h. Large-scale susceptibility testing using the interim discriminating concentration of 100 µg AI/bottle against thousands of *An. gambiae s.l.* in 16 countries showed that this concentration was generally suitable, with a mean of 98.7% mortality (95% CI: 98.1–99.3). Other studies in Faranah Prefecture of Guinea with 100 µg AI/bottle produced 100% mortality with wild *An. gambiae s.s*., while in the Agréby-Tiassa Region of south-east Côte d’Ivoire, the same concentration produced only 95.5% mortality [36, 37]. Using the formula of Lees et al. it was determined that 200 µg AI/bottle was a suitable discriminating concentration and this concentration is likely to produce fewer cases of false resistance reporting than with 100 µg AI/bottle. It is recognized that susceptibility testing with chlorfenapyr will produce more variable results than with neurotoxic insecticides due to the mode of action being linked with the metabolism of the insect, and this variability was most evident at lower concentrations. WHO recommend a temperature of 27 ± 2 °C be closely adhered to when conducting chlorfenapyr bioassays [32]. In this study (and probably in general) it was not always possible to closely regulate testing and holding temperature and this may have been a factor in the generally lower mortality in Madagascar. The activation of chlorfenapyr and its toxic action of disrupting cellular respiration, being metabolic processes, are both temperature dependent [32]. This is likely to lead to significant variation in test results between laboratories unless stricter temperature control measures are undertaken. In colder settings heaters should be used (especially overnight) to ensure testing and holding conditions always meet the minimum WHO recommended temperature of 25 °C. The higher potential for test variability highlights the need to repeat bioassays to confirm resistance over several time points, particularly when resistance is being reported for the first time. To minimize the occurrence of false resistance reporting, tests should always be conducted in parallel with a well-characterized colony strain to try and detect issues with under-dosing or low testing temperature. It is also recommend that before reporting chlorfenapyr resistance for a site, experiments should be repeated at the same location at least three times in different months and consistently result in less than 90% mortality. Ideally any findings of potential resistance would be supported by molecular studies (for example, transcriptome sequencing to identify upregulated and downregulated genes) to identify mechanisms of resistance.

Insecticide selection pressure from agriculture is generally regarded as an important early driver of insecticide resistance in malaria vectors [38, 39]. Chlorfenapyr resistance has been reported in several species of crop pest due to agricultural use in Asia, Europe, North America, and Australia, [40–42]. While statistics from sub-Saharan Africa are scant, there appears to be little usage of pyrrole insecticides for agricultural pest control, with supply by BASF limited to Kenya for control of rose pests in greenhouses (Dr S Stutz, BASF 2020, pers. commun.). However, it is likely that generic formulations will become more widely available, for example chlorfenapyr residues have already been detected on cabbages in Botswana [43]. Limited agricultural use of chlorfenapyr in sub-Saharan Africa would help to preserve susceptibility of the vector to this important insecticide by limiting selection pressure to public health use. The only report of potential chlorfenapyr resistance to date is from Côte d’Ivoire by Kouassi et al. which showed that 200 µg AI/bottle killed less than 98% of *An. gambiae s.l.* in five of 15 sites, with possible resistance recorded in Bouaké, Gagnoa, Nassian, Sakassou, and San Pédro [44]. This could be a sign of chlorfenapyr resistance in some parts of Côte d’Ivoire, but further phenotypic and genotypic data should be collected to confirm this finding. Fortunately, results from this study in 16 countries in sub-Saharan Africa have shown no signs of chlorfenapyr resistance.

Cluster-randomized control trials of Interceptor G2 ITNs in Tanzania and Benin are nearing completion, pilot distribution evaluations are underway in Burkina Faso, Rwanda, Mali, Mozambique, and Nigeria, and a MedAccess partnership has reduced the price of 35 million Interceptor G2 nets by 40% [45, 46], which is expected to greatly increase the availability of these products. With millions of Interceptor G2 nets being distributed, it is essential for a discriminating concentration to be determined and susceptibility testing to be regularly conducted to ensure there is no cross-resistance through existing mechanisms and to monitor any developing resistance. In a time where new insecticides are desperately needed, it is vitally important for timely susceptibility protocols for new active ingredients. Results from this study have been included as part of the WHO expert committee to determine a suitable discriminating concentration and a WHO recommendation is expected in 2021.

## Conclusion

This study showed that 100 or 200 µg AI/bottle chlorfenapyr in bottle bioassays are suitable discriminating concentrations for monitoring susceptibility of wild *An. gambiae s.l*., with mortality recorded up to 72 h. Testing in 16 countries in sub-Saharan Africa demonstrated malaria vector susceptibility to chlorfenapyr at all sites, including mosquitoes with multiple resistance mechanisms to pyrethroids.

## Supplementary Information

**Additional file 1: .**

**Additional file 2: .**

**Additional file 3: .**

**Additional file 4: .**

## Data Availability

All data analysed during this study are included in this published article and its supplementary information files.

## Abbreviations

AI: Active ingredient
ATP: Adenosine 5'-triphosphate
CDC: US Centers for Disease Control and Prevention
CI: Confidence interval
IQR: Interquartile range
IRS: Indoor residual spraying
ITN: Insecticide-treated nets
NMCP: National Malaria Control Programme
PMI: US President’s Malaria Initiative
PQ: Prequalification listing
WHO: World Health Organization

## References

[1] WHO. World Malaria Report 2019. Geneva, World Health Organization, 2019. https://www.who.int/publications/i/item/9789241565721

[2] WHO. World Malaria Report 2020: 20 years of global progress and challenges. Geneva, World Health Organization, 2020. https://www.who.int/publications/i/item/9789240015791

[3] Bhatt S, Weiss DJ, Cameron E, Bisanzio D, Mappin B, Dalrymple U (2015). The effect of malaria control on *Plasmodium falciparum* in Africa between 2000 and 2015. Nature.

[4] Long-lasting insecticidal net (LLIN) price data https://www.unicef.org/supply/reports/long-lasting-insecticidal-net-llin-price-data

[5] Zaim M, Aitio A, Nakashima N (2000). Safety of pyrethroid-treated mosquito nets. Med Vet Entomol.

[6] WHO. List of WHO Prequalified Vector Control Products. Geneva, World Health Organization, 2020. https://www.who.int/pq-vector-control/prequalified-lists/VCP_PQ-List_26August2020.pdf?ua=1

[7] Ranson H, Lissenden N (2016). Insecticide resistance in African *Anopheles* mosquitoes: a worsening situation that needs urgent action to maintain malaria control. Trends Parasitol.

[8] Sovi A, Keita C, Sinaba Y, Dicko A, Traore I, Cisse MBM (2020). *Anopheles gambiae (s.l.)* exhibit high intensity pyrethroid resistance throughout Southern and Central Mali (2016–2018): PBO or next generation LLINs may provide greater control. Parasit Vectors..

[9] Wat'senga F, Agossa F, Manzambi EZ, Illombe G, Mapangulu T, Muyembe T (2020). Intensity of pyrethroid resistance in *Anopheles gambiae* before and after a mass distribution of insecticide-treated nets in Kinshasa and in 11 provinces of the Democratic Republic of Congo. Malar J.

[10] Awolola TS, Adeogun A, Olakiigbe AK, Oyeniyi T, Olukosi YA, Okoh H (2018). Pyrethroids resistance intensity and resistance mechanisms in *Anopheles gambiae* from malaria vector surveillance sites in Nigeria. PLoS ONE.

[11] Pwalia R, Joannides J, Iddrisu A, Addae C, Acquah-Baidoo D, Obuobi D (2019). High insecticide resistance intensity of *Anopheles gambiae (s.l.) *and low efficacy of pyrethroid LLINs in Accra Ghana. Parasit Vectors.

[12] N'Guessan R, Odjo A, Ngufor C, Malone D, Rowland M. a chlorfenapyr mixture net Interceptor® G2 shows high efficacy and wash durability against resistant mosquitoes in West Africa. PLoS ONE. 2016. 11:e0165925. 10.1371/journal.pone.0165925 PMC5112870 27851828

[13] Bayili K, N’do S, Namountougou M, Sanou R, Ouattara A, Dabire RK (2017). Evaluation of efficacy of Interceptor((R)) G2, a long-lasting insecticide net coated with a mixture of chlorfenapyr and alpha-cypermethrin, against pyrethroid resistant *Anopheles gambiae s.l*. in Burkina Faso. Malar J.

[14] Camara S, Ahoua Alou LP, Koffi AA, Clegban YCM, Kabran JP, Koffi FM (2018). Efficacy of Interceptor® G2, a new long-lasting insecticidal net against wild pyrethroid-resistant *Anopheles gambiae* s.s. from Côte d'Ivoire: a semi-field trial. Parasite.

[15] IRAC Mode of Action (MOA) Classification Scheme https://irac-online.org/documents/moa-classification/

[16] Black BCHK, Ahmmadsahib CD, Kukel CD, Donovan S (1994). Insecticidal action and mitochondrial uncoupling activity of AC-303,630 and related halogenated pyrroles. Pesticide Biochem Physiol.

[17] Pesticide Fact Sheet Chlorfenapyr https://www3.epa.gov/pesticides/chem_search/reg_actions/registration/fs_PC-129093_01-Jan-01.pdf

[18] Chlorfenapyr (254) http://www.fao.org/fileadmin/templates/agphome/documents/Pests_Pesticides/JMPR/Evaluation12/Chlorfenapyr.pdf

[19] WHO. Test procedures for insecticide resistance monitoring in malaria vector mosquitoes, second edition. Geneva, World Health Organization, 2016.

[20] CDC. Guideline for evaluating insecticide resistance in vectors using the CDC bottle bioassay. In http://www.cdcgov/ncidod/wbt/resistance/assay/bottle/indexhtm

[21] WHO Pesticide Evaluation Scheme. Insecticide Resistance Monitoring In Disease Vectors Procedures and Conditions for Supply of Test Kits http://www.who.int/neglected_diseases/vector_ecology/resistance/WHO_Test_Kit_Catalogue_order_form_Oct2016.pdf.

[22] Gillies T, Coetzee M (1987). Supplement of the *Anopheles* of Africa south of Sahara (Afrotropical region). Publ S Afr Instit Med Res (Johannesburg).

[23] Santolamazza F, Mancini E, Simard F, Qi Y, Tu Z, della Torre A (2008). Insertion polymorphisms of SINE200 retrotransposons within speciation islands of *Anopheles gambiae* molecular forms. Malar J.

[24] Wilkins EE, Howell PI, Benedict MQ (2006). IMP PCR primers detect single nucleotide polymorphisms for *Anopheles gambiae* species identification, Mopti and Savanna rDNA types, and resistance to dieldrin in *Anopheles arabiensis*. Malar J.

[25] Scott JA, Brogdon WG, Collins FH (1993). Identification of single specimens of the *Anopheles gambiae* complex by the polymerase chain reaction. Am J Trop Med Hyg.

[26] Malaria Research and Reference Reagent Resource Center (MR4). Methods in *Anopheles* Research. 4th Edn. 2014.

[27] Lees R, Praulins G, Davies R, Brown F, Parsons G, White A (2019). A testing cascade to identify repurposed insecticides for next-generation vector control tools: screening a panel of chemistries with novel modes of action against a malaria vector. Gates Open Res.

[28] Report of the WHO Informal Consultation Test Procudures for Insecticide Resistance Monitoring in Malaria Vectors, Bio-Efficacy and Persistance of Insecticides on Treated Surfaces; WHO/CDS/CPC/MAL/98.12. https://apps.who.int/iris/handle/10665/64879

[29] Anto F, Asoala V, Anyorigiya T, Oduro A, Adjuik M, Owusu-Agyei S (2009). Insecticide resistance profiles for malaria vectors in the Kassena-Nankana district of Ghana. Malar J.

[30] Dagg K, Irish S, Wiegand RE, Shililu J, Yewhalaw D, Messenger LA (2019). Evaluation of toxicity of clothianidin (neonicotinoid) and chlorfenapyr (pyrrole) insecticides and cross-resistance to other public health insecticides in *Anopheles arabiensis* from Ethiopia. Malar J.

[31] Koenraadt CJ, Kormaksson M, Harrington LC (2010). Effects of inbreeding and genetic modification on Aedes aegypti larval competition and adult energy reserves. Parasit Vectors.

[32] Oxborough RM, N'Guessan R, Jones R, Kitau J, Ngufor C, Malone D (2015). The activity of the pyrrole insecticide chlorfenapyr in mosquito bioassay: towards a more rational testing and screening of non-neurotoxic insecticides for malaria vector control. Malar J.

[33] Rowland M (1989). Changes in the circadian flight activity of the mosquito *Anopheles stephensi* associated with insemination, blood-feeding, oviposition and nocturnal light intensity. Physiol Entomol.

[34] Meireles-Filho AC, Kyriacou CP (2013). Circadian rhythms in insect disease vectors. Mem Inst Oswaldo Cruz.

[35] Agumba S, Gimnig JE, Ogonda L, Ombok M, Kosgei J, Munga S (2019). Diagnostic dose determination and efficacy of chlorfenapyr and clothianidin insecticides against *Anopheles* malaria vector populations of western Kenya. Malar J.

[36] Stica C, Jeffries CL, Irish SR, Barry Y, Camara D, Yansane I (2019). Characterizing the molecular and metabolic mechanisms of insecticide resistance in *Anopheles gambiae* in Faranah. Guinea Malar J.

[37] Meiwald A, Clark E, Kristan M, Edi C, Jeffries CL, Pelloquin B, et al. Reduced long-lasting insecticidal net efficacy and pyrethroid insecticide resistance are associated with over-expression of CYP6P4, CYP6P3 and CYP6Z1 in populations of *Anopheles coluzzii* from South-East Côte d'Ivoire. J Infect Dis. 2020. (Online ahead of print) 10.1093/infdis/jiaa699 PMC9016462 33175129

[38] Hien AS, Soma DD, Hema O, Bayili B, Namountougou M, Gnankine O, Baldet T (2017). Evidence that agricultural use of pesticides selects pyrethroid resistance within *Anopheles gambiae* s.l. populations from cotton growing areas in Burkina Faso West Africa. PLoS ONE.

[39] Diabate A, Baldet T, Chandre F, Akoobeto M, Guiguemde TR, Darriet F (2002). The role of agricultural use of insecticides in resistance to pyrethroids in *Anopheles gambiae* s.l. in Burkina Faso. Am J Trop Med Hyg..

[40] Wang X, Wang J, Cao X, Wang F, Yang Y, Wu S (2019). Long-term monitoring and characterization of resistance to chlorfenapyr in *Plutella xylostella* (Lepidoptera: Plutellidae) from China. Pest Manag Sci.

[41] Herron GA, Rophail J, Wilson LJ (2004). Chlorfenapyr resistance in two-spotted spider mite (*Acari: Tetranychidae*) from Australian cotton. Exp Appl Acarol.

[42] Ullah F, Gul H, Desneux N, Said F, Gao X, Song D (2020). Fitness costs in chlorfenapyr-resistant populations of the chive maggot *Bradysia odoriphaga*. Ecotoxicology.

[43] Machekano H, Massamba W, Mvumi BM, Nyamukondiwa C (2019). Cabbage or ‘pesticide’ on the platter? Chemical analysis reveals multiple and excessive residues in African vegetable markets. Int J Food Contamination.

[44] Kouassi BL, Edi C, Tia E, Konan LY, Akré MA, Koffi AA (2020). Susceptibility of *Anopheles gambiae* from Côte d'Ivoire to insecticides used on insecticide-treated nets: evaluating the additional entomological impact of piperonyl butoxide and chlorfenapyr. Malar J.

[45] Joint News Release. BASF, MedAccess and the Bill & Melinda Gates Foundation collaborate to bring innovative mosquito nets to malaria-endemic countries https://www.basf.com/global/en/media/news-releases/2019/10/p-19-349.html

[46] The New Nets Project: Evidence base for new dual-AI nets https://www.ivcc.com/market-access/new-nets-project/

