# Supplementary material for: Determination of the discriminating concentration of chlorfenapyr (pyrrole) and Anopheles gambiae sensu lato susceptibility testing in preparation for distribution of Interceptor® G2 insecticide-treated nets
Source: Malar J. 2021 Jul 14;20:316. doi: 10.1186/s12936-021-03847-3 (PMC8278723; doi:10.1186/s12936-021-03847-3)
Supplement: Supplementary file 4 — Additional file 4: . [file 12936_2021_3847_MOESM4_ESM.docx]

**Table S1. Geographical location of sites in 10 countries where dose-range testing of five doses of chlorfenapyr was conducted with wild *An. gambiae* s.l., including information on the year that testing was conducted, resistance status to pyrethroids (PYR), carbamates (CARB), organophosphates (OP) and PCR species identification. Data sources for resistance status and species PCR are shown below.**

| Country | Region | Site | GPS co-ordinates | Year | Confirmed Resistance | | | Predominant vector species (identified by PCR) |
| --- | --- | --- | --- | --- | --- | --- | --- | --- |
|  |  |  |  |  | PYR | CARB | OP |  |
| DR Congo [1, 2] | Kinshasa | Kingasani | -04.404957  15.371890 | 2018 | ✓ | - | - | *An. gambiae* |
|  | Haut-Katanga | Kapolowe | -10.906717 26.946333 | 2018 | ✓ | - | - | *An. gambiae* |
|  | Tanganyika | Kalemie | -5.9192 29.18725 | 2018 | ✓ | - | - | *An. gambiae* |
|  | Bas-Congo | Kimpese | -5.574917 14.4285 | 2018 | ✓ | - | - | *An. gambiae* |
| Ethiopia [3] | Oromia | Abaya  (Sodere) | 8.399453 39.385231 | 2018 | ✓ | × | × | *An. arabiensis* |
| Ghana [4, 5] | Northern | Tamale  (Tugu) | 9.3722  -0.6068 | 2018 | ✓ | ✓ | × | *An. gambiae*/*coluzzii* sympatry |
|  |  | Bunkpurugu (Bunbuna) | 10.4499230.079017 | 2018 | ✓ | ✓ | × | *An. gambiae*/*coluzzii* sympatry |
|  |  | Kumbungu (Gbullung) | 9.486256  -1.012093 | 2018 | ✓ | ✓ | × | *An. gambiae*/*coluzzii* sympatry |
| Kenya [6] | Migori County | Rongo | -0.744121 34.603268 | 2017 | ✓ | × | × | *An. arabiensis* |
|  | Homa Bay County | Imbo | -0.59497 34.534661 | 2017 | ✓ | × | × | *An. arabiensis* |
| Madagascar [7] | Atsinanana | Brickaville (Ambodifaho) | -18.82191  49.06198 | 2018 | ✓ | × | × | *An. gambiae* |
|  | Analanjirofo | Fenereve Est (Mahambo) | -17.29000 49.28000 | 2018 | ✓ | × | × | *-* |
|  | Vatovavy Fitovinany | Vohipeno (Lanivo) | -22.35400  47.84030 | 2018 | × | × | × | *An. gambiae* |
|  | Analanjirofo | Vavatenina | -17.46669 49.19699 | 2018 | ✓ | × | × | *An. gambiae* |
|  | Atsianana | Toamasina (Vohitambato) | -18.14944 49.39860 | 2018 | ✓ | × | × | *An. gambiae* |
|  | Atsimo Atsinanana | Vondrozo (Mahatsinjo) | -22.805911 47.427513 | 2018 | × | × | × | *An. gambiae* |
|  | Vatovavy Fitovinany | Manakara (Ampasipotsy) | -22.129165 47.979347 | 2018 | × | × | × | *An. gambiae* |
|  | Atsimo Atsinanana | Vangaindrano (Lopary) | -23.34900 47.59650 | 2018 | × | × | × | *An. gambiae* |
|  | Atsimo Atsinanana | Manambotra Sud | -22.91670 47.81670 | 2018 | × | × | × | *An. gambiae* |
|  | Atsimo Atsinanana | Ampanihy (Manakaravavy) | -24.95900 44.26360 | 2018 | - | - | - | *-* |
| Mali [8, 9] | Mopti | Mopti | 14.512203  -4.19464 | 2017 | ✓ | × | × | *An.coluzzii* |
|  |  | Bankass | 14.090915 -3.524402 | 2017 | ✓ | × | × | *An. coluzzii* |
|  | Sikasso | Kadiolo | 10.557084 -5.75997 | 2019 | ✓ | - | × | *An. coluzzii* |
|  |  | Sélingué | 11.616667 -8.233333 | 2019 | ✓ | - | × | *An. coluzzii* |
|  | Kayes | Kayes | 14.492038 -11.50454 | 2019 | ✓ | - | × | *An. arabiensis* |
|  |  | Kita | 13.040198 -9.487983 | 2019 | ✓ | - | × | *An. gambiae* |
|  | Koulikoro | Kati | 12.744044 -8.066511 | 2019 | ✓ | - | × | *An. coluzzii* |
|  |  | Koulikoro | 12.86666 -7.56666 | 2019 | ✓ | - | × | *An. coluzzii* |
|  | Segou | Niono | 14.223544 -5.976456 | 2019 | ✓ | - | × | *An. coluzzii* |
|  |  | Bla | 12.95 -5.75 | 2019 | ✓ | - | × | *An. coluzzii* |
|  | Bamako | Bamako | 12.65001 -7.99986 | 2019 | ✓ | - | × | *An. coluzzii* |
| Nigeria [10] | Ebonyi State | Ohaukwu LGA | 6.47250 8.00290 | 2018 | ✓ | × | × | *An. gambiae*/*coluzzii* sympatry |
|  | Nasarawa State | Doma LGA | 8.40090 8.35810 | 2018 | ✓ | × | × | *An. gambiae* |
|  | Oyo State | Oluyole LGA | 7.20590 3.86550 | 2018 | ✓ | × | × |  |
| Senegal [11] | Dakar | Mbao | 14.74231 -17.33444 | 2018 | ✓ | ✓ | × | *An. arabiensis* |
|  | Kafrine | Koungheul | 13.98811 -14.68181 | 2018 | ✓ | × | × | *An. arabiensis*/*gambiae* sympatry |
|  | Tambacounda | Dianké Makha | 13.674167 -12.658333 | 2018 | ✓ | × | × | *An. arabiensis* |
| Uganda [12, 13] | Eastern Region | Soroti District | 1.73182 33.65378 | 2017 | ✓ | × | × | *An. arabiensis*/*gambiae* sympatry |
|  |  | Tororo District | 0.59898 34.06129 | 2017 | - | - | - | *An. arabiensis* |
| Zimbabwe | Midlands Province | Gokwe South | -17.7843 28.6264 | 2017 | - | - | - | *-* |

Key: ✓ resistance confirmed, - no testing conducted, × resistance not present.

**Data Sources**

1. Wat'senga F, Agossa F, Manzambi EZ, Illombe G, Mapangulu T, Muyembe T, Clark T, Niang M, Ntoya F, Sadou A, et al: **Intensity of pyrethroid resistance in Anopheles gambiae before and after a mass distribution of insecticide-treated nets in Kinshasa and in 11 provinces of the Democratic Republic of Congo.** *Malar J* 2020, **19:**169.

2. **The President’s Malaria Initiative (PMI) VectorLink Project TO1. The Democratic Republic of Congo Entomological Surveillance Final Report, December 1, 2017–December 31, 2018. Rockville, MD. Abt Associates.** [<https://www.pmi.gov/docs/default-source/default-document-library/implementing-partner-reports/drc-2018-entomological-monitoring-final-report.pdf>]

3. **PMI Vectorlink Ethiopia Project, Final Entomology Report, May 2018-April 2019, Rockville, MD. The PMI VectorLink project, Abt Associates Inc.** [<https://www.pmi.gov/docs/default-source/default-document-library/implementing-partner-reports/ethiopia_ento_final_report-_2018_submission3_11-8-19-sxf-(1).pdf?sfvrsn=4>]

4. **The PMI VectorLink Project. March 2019. Annual Entomological Monitoring Report for Northern Ghana, March 1-December 31, 2018. Abt Associates Inc.** [<https://www.pmi.gov/docs/default-source/default-document-library/implementing-partner-reports/ghana-2018-entomological-monitoring-final-report.pdf>]

5. **PMI Africa IRS (AIRS) Project Indoor Residual Spraying (IRS 2) Task Order Six. Entomological Monitoring of the PMI AIRS Program in Northern Ghana. 2017 Annual Report. Abt Associates Inc.** [<https://www.pmi.gov/docs/default-source/default-document-library/implementing-partner-reports/ghana-2017-entomological-monitoring-final-report.pdf>]

6. **AIRS Kenya Entomological Monitoring Annual Report. Rockville, MD. PMI | AFRICA IRS (AIRS) PROJECT INDOOR RESIDUAL SPRAYING (IRS 2) TASK ORDER SIX, Abt Associates Inc, January 4, 2018.** [<https://www.pmi.gov/docs/default-source/default-document-library/implementing-partner-reports/kenya-2017-entomological-monitoring-final-report.pdf>]

7. **The PMI VectorLink Project. September 2019. Madagascar Entomological Monitoring Final Report: June 2018 – May 2019. Rockville, Maryland. The PMI VectorLink Project, Abt Associates Inc.** [<https://www.pmi.gov/docs/default-source/default-document-library/implementing-partner-reports/508-compliant---pmi-vectorlink-madagascar-2018-entomological-monitoring-report.pdf>]

8. **The PMI Africa IRS (AIRS) Project Indoor Residual Spraying (IRS 2) Task Order Six. October 2017. AIRS Mali Entomological Monitoring Progress Report. Bamako, Mali. AIRS PROJECT, Abt Associates, Inc.** [<https://www.pmi.gov/docs/default-source/default-document-library/implementing-partner-reports/mali-2017-entomological-monitoring-final-report.pdf>]

9. **The PMI VectorLink Project Mali, Annual Entomological Monitoring Report. January - December 2019. Rockville, MD. The PMI VectorLink Project, Abt Associates Inc.** [<https://www.pmi.gov/docs/default-source/default-document-library/implementing-partner-reports/mali-2019-entomological-monitoring-final-report.pdf?sfvrsn=4>]

10. **The PMI VectorLink Project. April 2019. VectorLink Nigeria Final Entomology Report. April - September 2018. Rockville, MD. VectorLink, Abt Associates Inc.** [<https://www.pmi.gov/docs/default-source/default-document-library/implementing-partner-reports/nigeria-2018-entomological-monitoring-final-report.pdf?sfvrsn=5>]

11. **The PMI VectorLink Project. May 2019. Senegal Final Entomological Report: January 1, 2018 – February 28, 2019. Rockville, MD: The PMI VectorLink Project, Abt Associates Inc.** [<https://www.pmi.gov/docs/default-source/default-document-library/implementing-partner-reports/approved-pmi-vectorlink-senegal-2018-entomological-report_10-31-19-sxf.pdf>]

12. **The PMI VectorLink Project. Uganda Annual Entomology Report, December 1, 2017–December 31, 2018. Rockville, MD. Abt Associates.** [<https://www.pmi.gov/docs/default-source/default-document-library/implementing-partner-reports/uganda-2018-entomological-monitoring-final-report.pdf>]

13. **The PMI VectorLink Project Uganda. 2019. Addendum to Uganda Entomological Monitoring Annual Report, January 1 - December 31, 2018. Rockville, MD, USA: The PMI VectorLink Project, Abt Associates Inc.** [<https://www.pmi.gov/docs/default-source/default-document-library/implementing-partner-reports/uganda-2018-entomological-monitoring-final-report---addendum.pdf>]
